# Case report: Variability in clinical manifestations within a family with incontinentia pigmenti

**DOI:** 10.3389/fmed.2024.1402577

**Published:** 2024-07-17

**Authors:** Tatiana Belysheva, Tatiana Nasedkina, Irina Kletskaya, Dana Volchek, Irina Barinova, Vera Semenova, Aida Gadzhigoroeva, Ekaterina Zelenova, Timur Valiev, Elena Sharapova, Anna Michenko, Anastasiia Allenova, Darya Ponomareva

**Affiliations:** ^1^N.N. Blokhin National Medical Research Center of Oncology, Ministry of Health of Russia, Moscow, Russia; ^2^Engelhardt Institute of Molecular Biology of the Russian Academy of Sciences, Moscow, Russia; ^3^Russian Children's Clinical Hospital of Pirogov Russian National Research Medical University, Ministry of Health of Russia, Moscow, Russia; ^4^I.M. Sechenov First Moscow State Medical University (Sechenov University), Moscow, Russia; ^5^Central Research Institute of Dentistry and Maxillofacial Surgery, Moscow, Russia; ^6^Moscow Scientific and Practical Center of Dermatovenereology and Cosmetology, Moscow, Russia; ^7^Federal State Budgetary Institution "Central State Medical Academy" of the Administrative Department of the President of the Russian Federation, Moscow, Russia; ^8^Medical Scientific and Educational Center of Lomonosov Moscow State University, Moscow, Russia; ^9^International Institute of Psychosomatic Health, Moscow, Russia; ^10^The branch of Hadassah Medical LTD, Moscow, Russia

**Keywords:** incontinentia pigmenti, squamous cell carcinoma, dental abnormalities, hair, IKBKG/NEMO deletion, X-chromosome inactivation, family case report

## Abstract

Diagnosing skin diseases in children can be a complex interdisciplinary problem. Incontinentia pigmenti (IP), also known as Bloch-Sulzberger syndrome, is a rare hereditary genodermatosis related to a mutation in the *IKBKG* gene. We present a family case of IP described from the perspective of various specialists, including dermatologists, oncologists, geneticists, dentists, and trichologists. The peculiarity of this case is the development of squamous cell carcinoma (SCC) on the shin of a 10-year-old female patient with IP. The patient had a positive family history: her mother and two sisters also displayed clinical manifestations of IP with involvement of skin, teeth and hair. The presence of exons 4–10 deletion in the *IKBKG* gene in all affected females was confirmed by detailed genetic evaluation using long-range PCR, and also high degree of X-chromosome inactivation skewing was demonstrated. The family underwent a comprehensive examination and was followed up for 2 years with successful symptomatic treatment of dermatologic manifestations. Recommendations were also made regarding dental and hair problems. By the end of the follow-up period, patients had stabilized, with the exception of a 36-year-old mother who developed generalized morphea. The study demonstrates the varying expressiveness of clinical symptoms among family members and emphasizes the importance of timely diagnosis for effective management of patients with IP.

## 1 Introduction

Incontinentia pigmenti (IP) is a very rare dominant disease, with an incidence of 1.2 per 100,000 births, affecting skin and its derivatives (teeth, hair, nails, sweat and sebaceous glands), as well as other ectodermal tissues (central nervous system) (1). IP is caused by mutations in the *IKBKG* gene located on the X chromosome at locus Xq28. The encoded NEMO/IKKγ protein plays an important role in the activation of the NF-κB signaling pathway involved in immunity, inflammation, cell proliferation, and apoptosis (2). Hemizygous variants with loss of IKBKG/NEMO function are lethal in males, while IKBKG/NEMO variants with partial suppression of protein function are observed in heterozygous females with IP (3), as well as in males with the mosaic form of the disease or XXY genotype (Klinefelter syndrome) (4).

The disease usually starts in a few days after birth and looks like a neonatal skin infection (1, 5). The acute inflammatory process tends to subside and the initial vesiculobullous rash is replaced by a verrucous stage in postneonatal period, then progresses to a hyperpigmented stage that lasts until adolescence, and finally, to a hypopigmented or atrophic stage in adulthood (1, 6). Teeth and hair are also most often affected from infancy and childhood. Dental abnormalities include hypodentia or oligodentia, tooth shape disturbance (conical dystrophy) (6, 7). Manifestation in the scalp region presents as scarring alopecia and is seen in 28% (8) to 70% of cases (9). Eye abnormalities occur in every third patient and often manifest as microphthalmia, optic atrophy, retinopathy, cataract, pseudoglioma, and retrolental fibroplasia. In 33% of cases neurological manifestations, such as convulsive syndrome, spastic paraplegia are observed; in 16% of cases developmental disability is detected (1, 8).

A rather rare event in IP is painful subungual tumors, which usually manifest after puberty (10). Single cases of multiple aggressive subungual SCC or SCC arising on the skin of patients with IP have also been described, mainly in adult patients (11–13). In this report, we presented a family with four cases of IP (mother and her three daughters) with very rare manifestation of the disease, SCC developing on the shin of the eldest daughter at the age of 10 years. The diversity of clinical features within this familial case and implications for management of the patients with IP is discussed.

## 2 Case presentation

A 13-year-old girl (P) was referred to a dermatologist due to skin lesions on the front of her right leg. It was known from the medical history the presence of erythematous areas and pustules on her skin at birth. By the 8th day of life, rashes arose on her limbs, back, scalp, then vesicles began to disappear with the formation of erosions. The geneticist suspected IP, and hormonal therapy (prednisone) and topical treatment (salicylic zinc) were prescribed. Papules were observed on the skin up to 1.5 years of age, and then only areas of depigmentation persisted.

**Figure 1:**
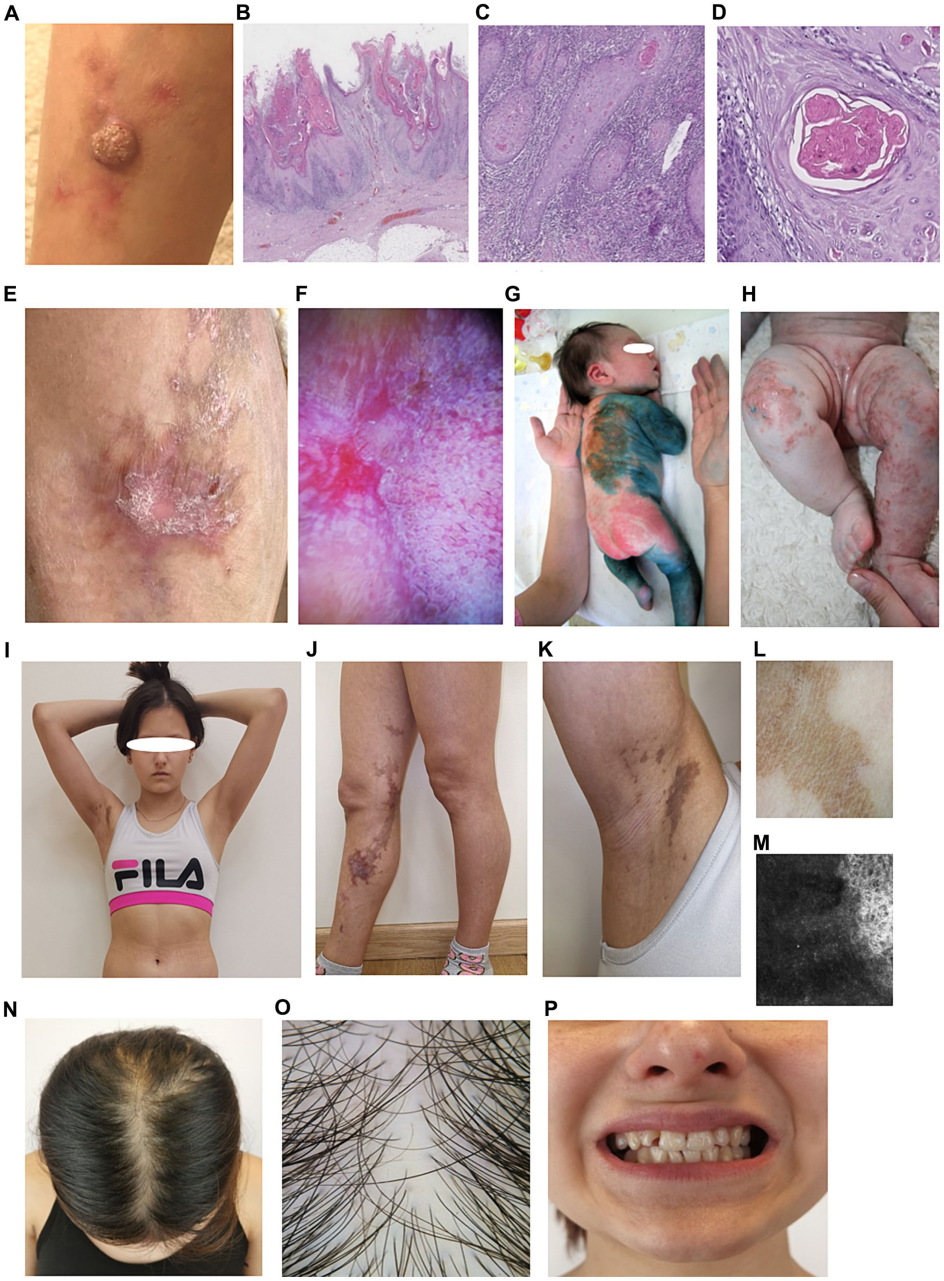
Phenotypic characteristics of the proband P. **(A)** Hyperkeratotic nodule on the skin of right shin. **(B)** Skin area with growth of an exophytic mass, epidermis with marked hyperkeratosis, verrucous papillomatosis and acanthosis, linear lichenoid lymphocytic infiltration (hematoxylin and eosin), x80. **(C)** Invasive complexes of neoplastic epithelium in the dermis, with dense lymphocytic infiltration, x200. **(D)** Concentric aggregations of the neoplastic epithelium with central keratinization, impaired maturation, moderate cytologic atypia, x350. **(E)** Сurrent state of the area of operation. **(F)** Dermatoscopy reveals Wickham streae (left) and pseudo follicular openings surrounded by violaceous halo (right), typical for hypertrophic lichen ruber planus. **(G,H)** First days of life. **(G)** Erythematous rush. **(H)** Vesicles and erosions on the patient skin. **(I–K)** Present state. **(I)** Overview. **(J,K)** Areas of hyperpigmentation involving inner leg and armpit. **(L)** Homogenous brown pigmentation is visible under dermoscopy (Dermlite 4, polarized mode). **(M)** Confocal microscopy reveals uneven melanin accumalation in hyperpigmented spots. **(N)** Parietal hair thinning, brownish discoloration of the scalp in previously affected areas. **(O)** Areas of scarring alopecia, single hairs predominate (Dermlite x 10, polarized mode). **(P)** Dental malformations.

At the age of 10 years, a rapidly growing hyperkeratotic nodular skin tumor appeared on the right shin. The firm nodule up to 2.5 cm in diameter with central hyperkeratotic masses, partially with hemorrhagic crust, fused with surrounding tissues and accompanied by periodic moderate itching. Six months later, wide excision of the nodule was performed with local tissue replacement of the defect. Histological examination revealed invasive well-differentiated keratinizing SCC (Figures 1A–D). A comprehensive analysis was performed to exclude tumor dissemination and the presence of metastatic lesions.

Two years after excision, the same area presented a linear rash distributed along Blaschko’s lines, with grayish macules in the upper third, interrupted by star-shaped red-brown lichenoid plaques in the operated area. *In vivo* confocal laser scanning microscopy confirmed lichen planus with partial disruption of the epidermis, dyskeratosis and hyperkeratosis, irregular acanthosis, inflammatory infiltration, hypergranulosis, dilated vessels, and fibrosis (Figures 1E,F). Detailed phenotypic characteristics of P are presented on Figures 1G–P. The family history revealed the presence of characteristic skin defects, dental and hair abnormalities in other female relatives: mother and two sisters. Detailed examination was done for all affected family members.

**Figure 2:**
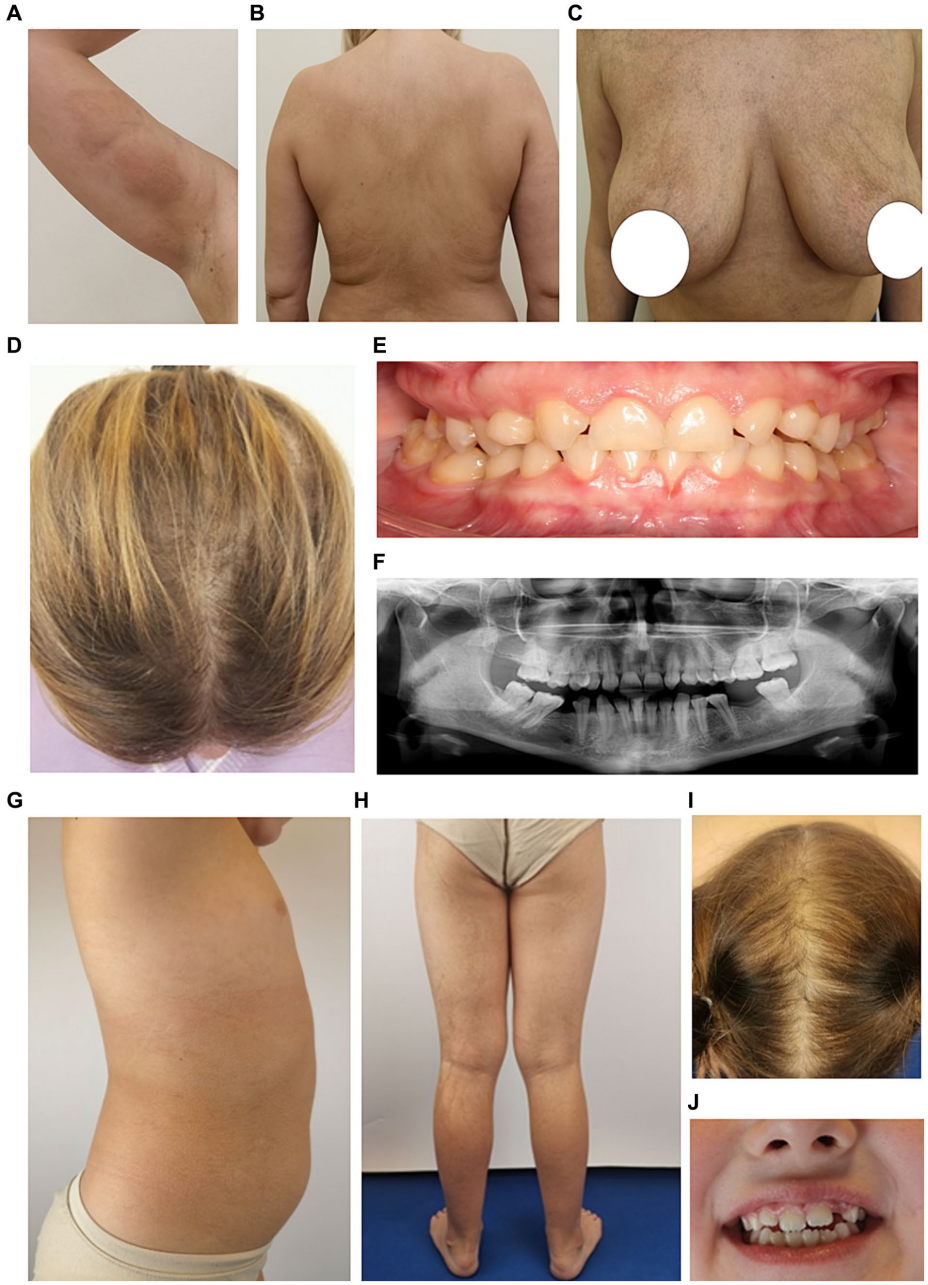
Phenotypic characteristics of the proband’s mother M **(A–F)** and sister S1 **(G–J)**. **(A–C)** Areas of irregular pigmentation in M. **(D)** Parietal zone with hair thinning. **(E)** Dental malocclusion (adentia of both upper lateral insicors and also two molars on the lower jaw are missing). **(F)** Orthopantomogram shows anomalies in the positioning of individual teeth, adentia of both maxillary lateral insicors and two molars on the lower jaw. **(G,H)** Areas of hyperpigmentation on the trunk and legs in S1. **(I)** Parietal zone with sparse hair growth. **(J)** Mild dental malformations.

A 34-year-old mother (M) presented in infancy with erythematous patches and pustules on the skin, later replaced by papules and vesicles. During the first year of life, she was periodically admitted to hospital with the following diagnoses: herpetic infection, erythema multiforme, and lichen planus. Our examination revealed rounded pigmented atrophic patches on the skin of the right shoulder, chest, and back, as well as linear areas of hypopigmentation with mild skin atrophy on the lower extremities. Moderate thinning of hair in the parietal region, and dental malocclusion were observed (Figures 2A–F). Obstetric history revealed five pregnancies: three ended in delivery, one in miscarriage at 12 weeks, and the fifth pregnancy was medically terminated.

A 7-year-old girl (S1) is the second child in the family. At birth, a widespread vesicular, erythematous rash was observed on the skin. Local therapy with antiseptic solutions was performed. By the age of 1 year, the rash had resolved, and only areas of hyperpigmentation persisted. At present, the parietal zone with sparse hair growth and mild dental malformations were observed (Figures 2G–J).

**Figure 3:**
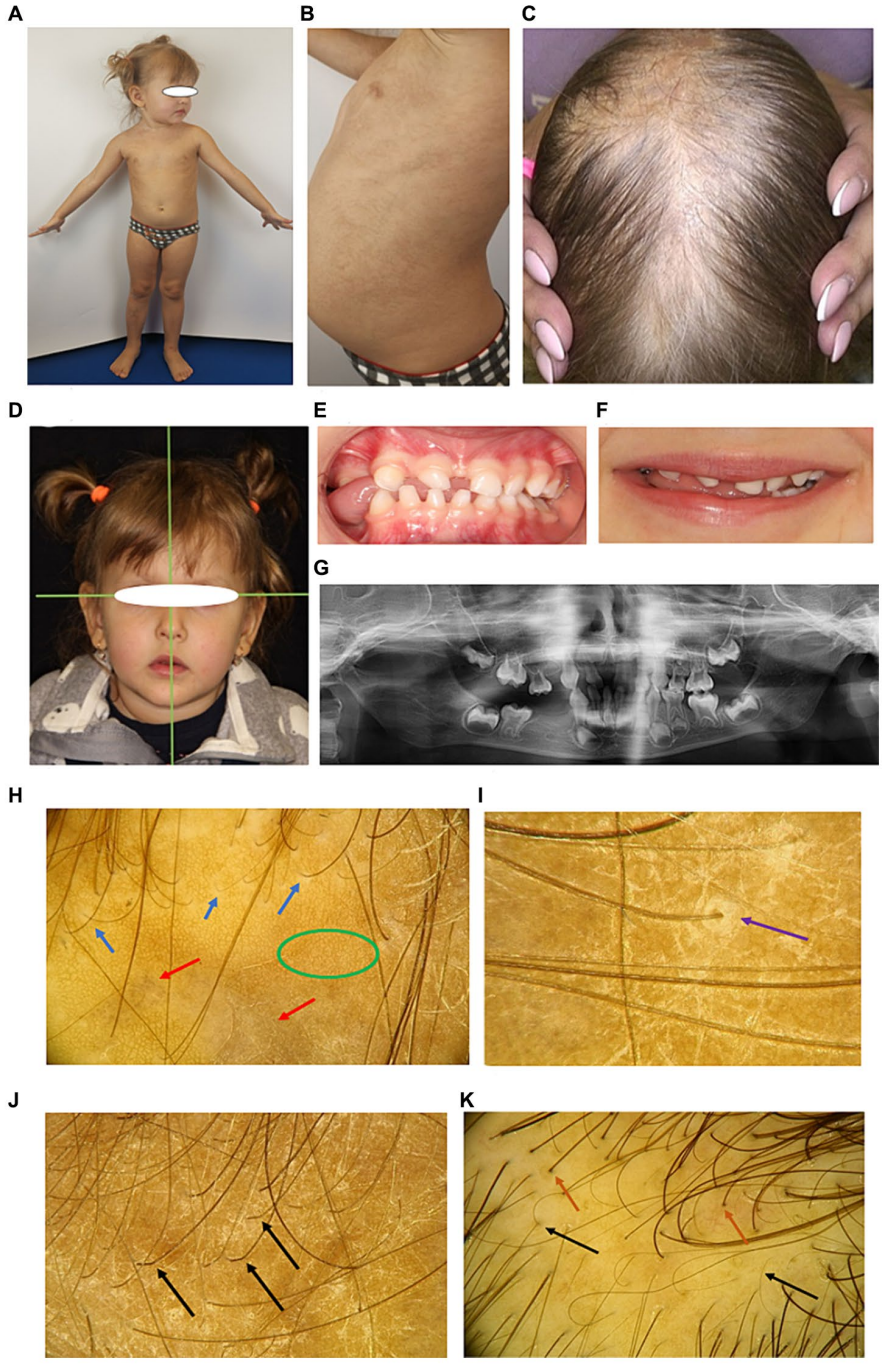
Phenotypic characteristics of proband’s sister S2 **(A–G)** and trichoscopic hair evaluation of family members **(H–K)**. **(A)** Overall view. **(B)** Light brown linear, curved and “star-like” macules are preserved on the trunk. **(C)** Severe alopecia in parietal zone. **(D)** Compensatory tilt of the head to the left, corners of the mouth downwards, lips are not closed in the resting position. **(E)** Laying of the tongue between the teeth on the right side in the area of missing teeth. **(F)** The smile is asymmetrical, the tongue is inserted between the teeth on the right side. **(G)** Orthopantomogram reveals multiple tooth adentia. **(H)** Trichoscopy of the scalp of S1 and S2 demonstrates growth of mostly single hair, white peripilar dots (blue arrows), honeycomb-shaped pigmentation (green oval), and scalp discoloration (red arrows). **(I)** Keratinous perifollicular desquamation (purple arrow). **(J)** Boomerang-type hair bending in the proximal zone of growing single hair (shown by arrows). **(K)** A large number of single growing hair (brown arrows) and the presence of vellus hair (black arrows) on the scalp of M.

A 3-year-old girl (S2) is the third child in the family. At birth, she had a widespread vesicular, erythematous rash on the skin. Topical therapy was successful, but by 9 months of life, after remission, the vesicular skin rash reappeared. The whole process was wavy and the most severe compared to other affected members. On examination, “star-like” pigment spots were observed on the skin in the axillary region, trunk, groin area, shins and back. The hair loss on the vertex of scalp was obvious. The most pronounced dental malformations compared to other family members were identified. The absence of temporary teeth resulted in a change in facial configuration by reducing the height of the lower third of the face. Compensatory contraction of facial muscles led to functional remodeling, which could be observed when smiling or talking, and the habit of placing the tongue over the area of the dental defect was developed. Orthopantomogram reveled the absence of several deciduous teeth (lactodentia; Figures 3A–G).

Detailed examination of scalp and hair conditions of the family members demonstrated the loss of follicular orifices. White peripilar dots were identified in S1 and S2, but not in M and P, indicating stabilization of the scarring process with age. Both S1 and S2 exhibited lamellar microshealing characteristic of the subacute stage of squamous lichen planus, and the proximal portions of the hair shafts had a boomerang-like bend. Discoloration, honeycomb pigmentation, and single hair growth were characteristic for all females; M had an increased number of vellus hairs on the vertex, indicating miniaturization of hair follicles, possibly due to their own sensitivity to androgens (Figures 3H–K).

**Figure 4:**
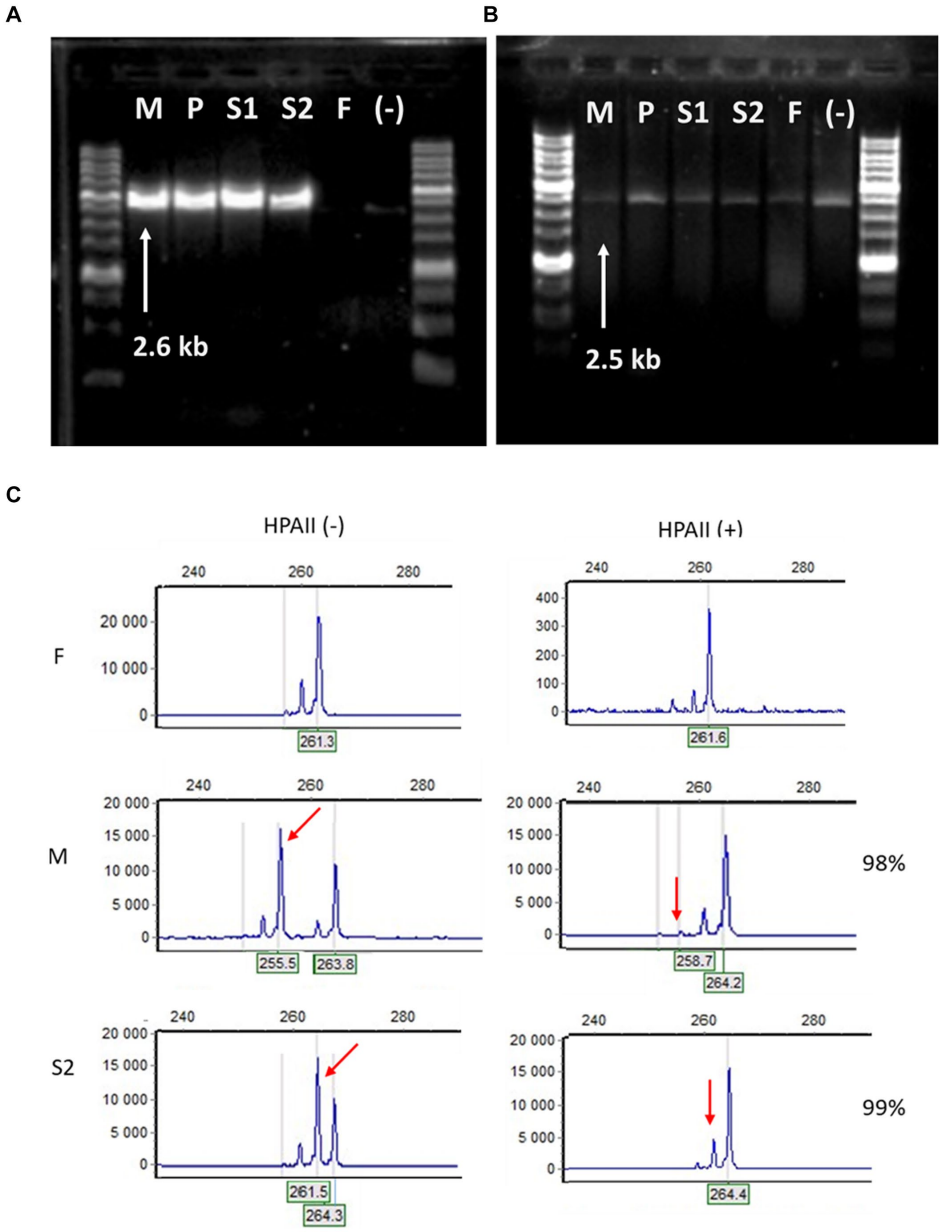
Molecular-genetic analysis. **(A)** Long-range PCR with *IKBKG* primer pair In2/JF3R revealed IP-specific 2.6-kb band in patient samples (M – mother, P – proband, S1 – sister 1, S2 -sister 2) and the absence of PCR-product in healthy people (F – father of the family and (−) – negative control). **(B)** In contrast, bands amplified with primers specific for *IKBKGP* (JF3R/Rev2) showed no appreciable specificity between patients and controls. **(C)** Fragment analysis of PCR products amplified from undigested HPAII (−) and digested HPA (+) DNA of F, M and S2. In the F DNA, one major peak decreases significantly after HpaII digestion. In the undigested M DNA, the two major peaks represent two alleles with different numbers of short tandem repeats at the *AR* locus, after digestion the short allele is practically disappeared (red arrow). The S2 DNA displays the preferential loss of the active father’s (F) allele (red arrow), while the inactivated M allele is preserved.

Genomic DNA isolated from peripheral blood was used in genetic studies. A long-range PCR was performed with primer pairs specific for the *IKBKG* gene and the *IKBKGP* pseudogene, as previously described (14). The deletion of exon 4–10 of *IKBKG* gene was present in mother and three daughters, while absent in father and healthy control (Figures 4A,B). To examine the skewing of X-chromosome inactivation (XCI), the methylation pattern of the repeat region (CAG)_n_ in exon 1 of the *AR* gene was investigated in the affected females using a HUMARA assay (15). All females had unbalanced inactivation of the X chromosome (> 90%) in peripheral blood leukocytes with the following values: P and M had 98%, S1 had 96%, and S2 had 99% of skewing (Figure 4C).

The family was consulted and then followed-up by multidisciplinary team for the last 2 years. Recommendations on symptomatic and compensatory treatment aimed at stabilization of pathological processes were given. To solve skin problems of P, topical treatment of lichen planus with clobetasol propionate for 2 weeks with further tapering was prescribed. Soon, the rashes smoothed and faded, but did not disappear completely, so emollients were recommended as supportive therapy. Further, the flattening of the lichen planus lesions and partial regression of hyperpigmented papules was noted. For M, S1 and S2 having minor skin lesions, supportive care with sun protection and emollients was recommended, as well as avoiding trauma and keeping the skin cool and dry. The status of S1 and S2 remained stable throughout the follow-up period. In a year and a half, M noted the appearance of multiple asymptomatic skin lesions that rapidly spread to the skin of the abdomen, upper and lower extremities, and the gluteal region. A diagnosis of generalized morphea was established. Systemic treatment with penicillin, pentoxifylline, bovhyaluronidaze azoximer was used to stabilize the disease.

To alleviate dental problems, M. was recommended prosthetics in the area of missing teeth, periodontal treatment and rehabilitation. The treatment of children with several congenitally missing teeth is challenging, especially when combined with malocclusion, because growth and development of the oral structure must be considered at the same time. Individualized treatment plans for P, S1, and S2 with missing teeth were made based on a comprehensive assessment of age and the need to restore occlusal and chewing function, as well as esthetic requirements for tooth shape and alignment.

To stabilize secondary scarring alopecia, betamethasone treatment was offered to younger daughters S1 and S2, as the scalp trichoscopy revealed mild perifollicular hyperkeratosis. This sign indicates the persistence of an active pathological process in the lesions. In the older family members, M and P, no signs of active hair loss were revealed, so they were offered hair transplantation or trichopigmentation to camouflage baldness.

## 3 Discussion

The penetrance of IP is 100%, but the expressiveness of clinical manifestations can vary widely between patients, even within the same family (8). When analyzing the clinical data of 381 patients from the IP Genetic Biobank, it was shown that erythematous-vesicular rash in the neonatal period is the most characteristic feature and occurs in 90% of patients. Transition to the verrucous stage within first year of life was observed only in 46% of patients, while the third stage with hyperpigmentation again was described in the majority of patients (85%). The fourth hypopigmentation stage is observed in 20% of patients, with the age of manifestation ranging from childhood to adults (1, 16, 17). In our case, characteristic skin lesions at the first erythematous stage were observed in all family members, but the second stage was absent, at least in S1. In M, areas of hypopigmentation and linear skin atrophy were noted in adulthood; in the daughters, hypopigmented areas were observed from 1.5 years of age. Thus, omission or superimposition of different stages of skin lesions is quite common and can lead to blurring of the clinical picture and underdiagnosis of the disease.

In most cases, skin lesions disappear completely with age. Nevertheless, benign (subungual tumors) and malignant skin neoplasms may develop in individual cases (10–13). In our case, P was diagnosed with SCC on the skin of the right leg at the age of 10 years. Extremely rare cases of SCC have been described previously in adult patients (11), but only one case of SSC on the skin of the leg in a 16-year-old female patient (12). Also, basal cell carcinoma (BCC) was described in a 22-year-old female with IP (18).

SCC and BCC are non-melanoma skin cancer (NMSC) with similar pathogenesis: SCC develops through malignant proliferation of epidermal keratinocytes, while BCC arises from basal cells (19–22). Moreover, basosquamous carcinoma is described, which is characterized by a combination of clinical, dermoscopic, and histologic features from both BCC and SCC, as well as the presence of a transition zone (23). The malignization of keratinocytes may be associated with ultraviolet radiation, HPV infection, RAS/RAF/MEK/ERK and PI3K/AKT pathways (24). The development of malignant neoplasms in IP cases may be explained by inactivation of the NF-kB pathway due to *NEMO* gene mutation, which may promote uncontrolled cell proliferation (25, 26).

Treatment options of NMSC include surgery, Mohs micrographic surgery, curettage and electrodessication, radiation, photodynamic therapy, immunotherapy, topical (5-fluorouracil, imiquimod) and systemic therapy (chemotherapy, epidermal growth factor receptor inhibitors, hedgehog pathway inhibitors) (21, 27). Surgical excision alone leads to successful treatment of most NMSCs, and the cure rate is over 90% (28). In our case, only radical surgery was performed, and there was no recurrence of SCC during 5-year follow-up, but persistent inflammation at the surgical site requires continuous monitoring (19).

The frequency of dental anomalies in patients with IP ranges from 50 to 80% in different studies (9, 29, 30). Conical teeth, hypodentia (absence of one or more teeth) and oligodentia (absence of 6 or more teeth), and delayed tooth eruption are the most common manifestations. The population incidence of hypodontia of permanent teeth ranges from 2.7 to 12.2% in different ethnic groups (31), while the prevalence of dental hypo-and oligodentia in patients with IP is significantly higher, 31.2% (30). At the same time, adentia is a genetically heterogeneous phenomenon: both nonsyndromal hereditary forms caused by mutations in the genes *MSX1*, *PAX9*, *LTBP3*, *EDA*, and those associated with various hereditary syndromes have been identified (31).

In our study, hypodentia and anomalies of tooth arrangement were detected in M; also, serious abnormalities were found in P (delayed eruption of teeth, absence of a number of primary teeth, absence of 12 permanent teeth, conical teeth) and in S2 (prominent lactodentia). It should be noted that characteristic and pronounced dental anomalies are considered as an independent diagnostic criterion for the diagnosis of IP (6), nevertheless, this criterion is still more correctly applied in groups of patients with sick first-line relatives. The absence of temporary teeth in early childhood leads to the development of various functional disorders, which negatively affects the physical and psycho-emotional development of the child. Orthopedic and orthodontic care is provided to such children from 2.5–3 years of age in order to normalize the function of mastication, swallowing, speech, position of the mandible and tongue, and continues until the age of 18 years, when planning and carrying out permanent prosthetics is possible. The priority of complex early dental treatment of patients with missing teeth is restoration of masticatory function with removable plate prosthesis, and later with implantation (32).

In about 38% of patients with IP, alopecia on the vertex of the head is mild and goes unnoticed; this is probably why scarring alopecia retains its place in the minor criteria of the IP (5, 6). Agenesis or hypoplasia of the eyebrows and eyelashes is even rarer (8). On the other hand, scarring alopecia could be used as a marker to identify adult women affected with IP as older patients may have minimal cutaneous manifestations (33). In our family case, trichoscopic imaging showed the hallmarks of scarring alopecia on the skin and hair of the scalp. Discoloration, honeycomb pigmentation and absence of follicular orifices were found in both the mother and her daughters. White dots around follicles, characteristic of the period of destruction foci formation, as well as peripilar keratin desquamation were determined in S1 and S2, in which the period of scarring alopecia formation was shorter. A boomerang-type hair bend was detected in the proximal zone of growing single hairs. In general, hair auto transplantation can be considered to cover the most pronounced areas of alopecia when the pathological process on the scalp stabilizes with age.

The most common genetic mutation in IP is a deletion of exons 4 to10 in the *IKBKG* gene, which occurs in 65–80% of patients in different ethnic groups worldwide (2, 14, 34). Other types of mutations in the *IKBKG* gene have also been identified (point mutations, small insertions/deletions, and splice site mutations) (35). In our case, the disease is caused by the deletion of exon 4–10 of *IKBKG* gene, which results in the loss of large part of nucleotide sequence and, consequently, protein function. At the same time, clinical features among family members vary markedly in severity of manifestation. The phenomenon of X-chromosome inactivation has been shown to contribute significantly to the variability of clinical features (36). In our case, all affected members have unbalanced X-chromosome inactivation with XCI scores ranging from 96 to 99%, so the differences observed between patients are likely to be explained by other molecular mechanisms. The limitation of our study is that the follow-up is confined to short period and we could not follow the clinical presentation from birth to present for all family members.

In the neonatal period, the differential diagnosis of IP and skin infection can be made according to a number of clinical features: female gender, location of the rash along Blaschko’s lines, staging of skin manifestations, extensiveness of the lesion, persistent eosinophilia, lack of effect from antibacterial therapy, and absence of infectious history. In older age, special attention should be paid to patients with congenital adentia, abnormalities of the shape and location of teeth, and with hair problems. In our case, the mother had all characteristic skin lesions during first year of the life, but no proper diagnosis was established. Only after the birth of the first affected child, the family suspected and then confirmed the presence of IP. Despite this, the mother was not counseled by a geneticist about carrying the mutation and the possibility of passing this defect to her children, resulting in the birth of two more sick girls and one miscarriage. Accurate diagnosis of IP requires molecular genetic testing. But also, medical and genetic counseling in the family is very important both for pregnancy planning and for further treatment and dispensary observation of the mother carrying the mutation.

## 4 Conclusion

From a clinical standpoint, it is imperative to distinguish IP from other dermatologic conditions, because cells containing the mutated gene remain in the body after the lesions have repaired and further progression of the disease may occur. In the family described, one daughter developed SCC, so oncologic awareness should be ensured in patients with IP. In adulthood, the mother developed generalized morphea, which may be another clinical manifestation of the IP. Proper diagnosis and management of patients with IP requires a multidisciplinary approach involving physicians from different specialties.

## Data availability statement

The raw data supporting the conclusions of this article will be made available by the authors, without undue reservation.

## Ethics statement

The studies involving humans were approved by Local Ethics Committee of the N.N. Blokhin National Medical Research Center of Oncology of the Ministry of Health of the Russian Federation. The studies were conducted in accordance with the local legislation and institutional requirements. Written informed consent for participation in this study was provided by the participants’ legal guardians/next of kin. Written informed consent was obtained from the individual(s), and minor(s)’ legal guardian/next of kin, for the publication of any potentially identifiable images or data included in this article.

## Author contributions

TB: Conceptualization, Funding acquisition, Project administration, Writing – original draft. TN: Conceptualization, Supervision, Writing – original draft. IK: Data curation, Investigation, Writing – original draft. DV: Data curation, Methodology, Visualization, Writing – original draft. IB: Investigation, Methodology, Visualization, Writing – original draft. VS: Investigation, Methodology, Writing – original draft. AG: Data curation, Investigation, Visualization, Writing – original draft. EZ: Investigation, Validation, Writing – original draft. TV: Formal analysis, Writing – original draft. ES: Data curation, Validation, Writing – original draft. AM: Data curation, Investigation, Methodology, Visualization, Writing – original draft. AA: Data curation, Investigation, Validation, Writing – original draft. DP: Data curation, Formal analysis, Project administration, Validation, Writing – original draft.
